# Utilizing the Cool-Down Phenomenon to Differentiate Supraventricular Tachycardias in a Patient with COVID-19 Infection

**DOI:** 10.3390/clinpract11030068

**Published:** 2021-08-17

**Authors:** Khalid Sawalha, Vikram Baldini Gondhalekar, Nathan Klammer, Fuad Habash, Hakan Paydak

**Affiliations:** 1 Internal Medicine Division, White River Health System, Batesville, AR 72501, USA; 2 College of Medicine, University of Arkansas for Medical Sciences, Little Rock, AR 72002, USA; vbgondhalekar@uams.edu (V.B.G.); ngklammer@uams.edu (N.K.); 3 Cardiology Division, University of Arkansas for Medical Sciences, Little Rock, AR 72002, USA; fjhabash@uams.edu; 4 Electrophysiology Division, University of Arkansas for Medical Sciences, Little Rock, AR 72002, USA; HPaydak@uams.edu

**Keywords:** supraventricular tachycardia, cool down phenomenon, narrow complex tachycardia, encephalopathy, COVID-19 infection

## Abstract

A 63-year-old male patient with a history of hypertension, diabetes mellitus type 2, prostate cancer and class two obesity was admitted for encephalopathy. During his hospital stay he developed narrow complex tachycardia and it was difficult to definitively diagnose the underlying arrhythmia. Observation of the cool down phenomenon on telemetry strip allowed us to make the diagnosis of atrial tachycardia and elegantly rule out other causes. We report this interesting case of narrow complex tachycardia.

## 1. Introduction

Atrial tachycardia (AT) is a regular atrial rhythm at a constant rate of >100 beats per minute originating from outside of the sinus node [1,2]. Focal ATs arise from a single site within the left or right atrium, in contrast to reentrant atrial arrhythmias such as atrial flutter and atrial fibrillation, which involve multiple sites or larger circuits. 

AT have traditionally been characterized as automatic, triggered, or reentrant. However, the European Society of Cardiology and the North American Society of Pacing and Electrophysiology in 2001 proposed a classification that takes into consideration both anatomic features and electrophysiologic mechanisms [1]. AT is the overriding term that includes two major categories: 

(1) Focal atrial tachycardia due to an automatic, triggered, or micro-reentrant mechanism. (2) Macro-reentrant atrial tachycardia, including typical atrial flutter and other well-characterized macro-reentrant circuits in the right and left atrium. Intra-atrial reentrant tachycardia (IART) falls into the latter group. Furthermore, the joint American College of Cardiology/American Heart Association/Heart Rhythm Society 2015 guidelines further defined macro-reentrant atrial tachycardias that do not involve the tricuspid valve isthmus as “atypical or non-cavo-tricuspid isthmus-dependent atrial flutter” [3]. These macro-reentrant supraventricular tachycardias often involve the left atrium, particularly after atrial fibrillation (AF) ablation or Maze surgery for AF. They may involve any atrium where a scar, surgical or catheter-induced, may have taken place. 

Warm-up and cool-down phenomena refer to an observable acceleration and deceleration in heart rate, respectively, when transitioning from tachycardia to a normal rate. The presence of this suggests that enhanced automaticity underlies the tachyarrhythmia [1]. We report this interesting case of the cool down phenomenon in atrial tachycardia and its differential diagnosis.

## 2. Case Presentation

This is a 63-year-old man with a past medical history significant for hypertension, diabetes mellitus type 2, prostate cancer and class two obesity (BMI 38.9). He presented to an outside hospital with altered mental status. Magnetic resonance imaging (MRI) of the brain at the outside facility was negative, and he was transferred to our hospital for further evaluation. After arriving to our facility, the patient’s mental status was altered to the point that he was intubated in the emergency department. Lab work (including blood, urine and cerebrospinal fluid studies) were unremarkable, and in-house computed tomography angiography and MRI were negative for acute intra-cranial findings. The patient was transferred to the ICU for further investigation of encephalopathic processes and continued supportive therapy. 

On day 2 of admission, the patient converted from sinus tachycardia to non-sinus supra-ventricular tachycardia. The cardiology service was consulted at this time. The new rhythm was felt to be atrial flutter vs. focal atrial tachycardia, AVNRT, AVRT. There was no evidence for acute coronary syndrome or novel electrolyte/acid-base disturbances, and the arrhythmia was felt to be caused by increased sympathetic tone brought on by the patient’s critical condition. Ultimately, the cardiology service recommended rate control with an esmolol drip and monitoring of the rhythm for any changes. 

The subsequent hospital course lasted several weeks and was complicated, though less remarkable from a cardiologic point of view. The patient’s mental status has improved somewhat by day 3, and he was extubated. He was worked up in the hospital for his altered mental status, though a definitive diagnosis was never made. On Day 10, he was re-intubated for vocal cord paralysis. A few days after this, he was diagnosed with COVID-19. He spent 2 weeks in the dedicated COVID ICU and required tracheostomy and percutaneous endoscopic gastrostomy tube placement (PEG). His clinical condition improved by week 5 of admission. He was taken off the ventilator, and his PEG and urinary catheter were removed. He was transferred to the general hospital floor. He was later diagnosed with methicillin-susceptible Staphylococcus aureus (MSSA) bacteremia and started on appropriate antibiotics. However, his overall condition continued to improve, and he was transferred to a long-term care facility to complete his antibiotic course. The tracheostomy was left in place out of concern for continued vocal cord paralysis and was to be managed by the ear–nose–throat (ENT) service in the out-patient setting. Upon follow up, patient was doing well and remained in sinus rhythm. 

Throughout this hospital course, the patient’s cardiac rhythm switched back and forth between sinus and non-sinus supraventricular rhythm. Overall, adequate rate control was achieved with esmolol, initially, and later with a combination of metoprolol and diltiazem once the patient was able to tolerate oral medications. He eventually converted definitively into a normal rate and sinus rhythm prior to being discharged. The electrophysiology team reviewed the patient’s ECG data, and definitive identification of the arrhythmia is deliberated below. 

## 3. Discussion

**Figure 1:**
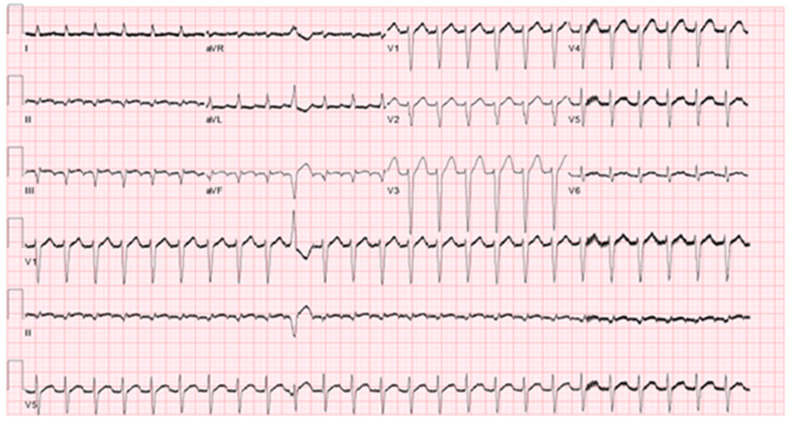
Electrocardiogram two hours later after the first ECG showing long RP supraventricular tachycardia with premature ventricular complexes delineating the p waves. However, cannot rule out atrial flutter with 2 to 1 block. Differential diagnosis includes atypical fast-slow AVNRT, orthodromic AVRT and atrial tachycardia. Q waves are also noted in leads II, III, AVF suggestive of old inferior myocardial infarction.

The differential diagnosis of the patient’s supra-ventricular tachycardia includes atrial flutter, atrio-ventricular nodal reentry tachycardia (AVNRT), focal atrial tachycardia and atrio-ventricular reentry tachycardia (AVRT). An ECG exhibiting the arrhythmia is demonstrated in Figure 1. 

The diagnosis of typical atrial flutter was strongly considered. Typical atrial flutter is caused by a macro-reentry circuit bound by the cavotricuspid isthmus inferiorly and the right atrial roof or the supero-posterior right atrium [4]. This produces an atrial rate of 240–350 bpm and is usually accompanied by a 2:1 AV block with a ventricular response of 120–150 bpm and a short R-P interval [5]. In the case presented, the patient had a regular supra-ventricular tachycardia with a ventricular rate of 150 bpm as seen in the electrocardiogram in Figure 1 which also shows a premature ventricular beat with no obvious P wave preceding this. In the case of atrial flutter, we would expect to find an atrial signal (flutter wave). There is no evidence of this. While this does not definitively rule out atrial flutter, it makes this diagnosis less likely.

AVNRT (typical and atypical) was also strongly considered. AVNRT is caused by a re-entry circuit localized to the AV node and produces a narrow complex tachycardia with absent or retrograde P-waves [6]. There are small but appreciable negative deflection after each T-waves in the inferior leads. These may represent retrograde atrial depolarization induced by a nodal re-entry circuit, but they could simply be part of the T-wave. Moreover, observation of a retrograde P-waves does not rule out a low atrial origin of the arrhythmia. 

Up to 20 percent of patients with AVNRT have uncommon forms of the arrhythmia, referred to as “atypical AVNRT” such as, antegrade conduction that can occur down the fast pathway with retrograde conduction up the slow pathway referred to as “fast-slow” AVNRT [7], multiple slow pathways resulting in “slow-slow AVNRT” variants in which both the antegrade and retrograde limbs of the circuit utilize slow AV nodal pathways, and rarely during AVNRT, conduction through the reentrant circuit is so slow that the heart rate is less than 100 beats per minute, by definition not a tachycardia. Despite the absence of tachycardia, patients can be symptomatic and may be treated with a slow pathway ablation. This arrhythmia, sometimes referred to as AV nodal reentrant arrhythmia (AVNRA), has been mistaken for a junctional rhythm.

Orthodromic AVRT is a macro-reentry circuit passing antegrade through the AV node and His-Purkinje system and retrograde through an accessory pathway [4]. This was also considered in the differential. The argument for and against this diagnosis is similar to that of AVNRT. We would expect a negative P-wave representing retrograde atrial depolarization to be present, though the presence of this is unclear and would not rule out atrial tachycardia from a near junctional origin. 

Focal atrial tachycardia was also considered. Atrial tachycardia is caused by one of several etiologies, including enhanced automaticity, triggered potential, or micro-reentry circuit [1]. The presence of discernable P-waves, a long R-P interval and an isoelectric baseline between atrial deflections differentiates atrial tachycardia from other causes of supraventricular tachycardia in most cases [4]. The presence of these features was difficult to confirm or deny due to the frequent ventricular repolarization/depolarization. 

**Figure 2:**
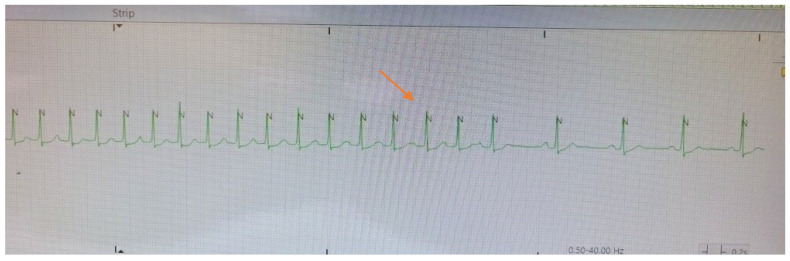
Telemetry strip showing resumption of normal sinus rhythm in the middle of the tracing at a rate of 126 bpm. The arrow draw attention to the cool down phenomenon before termination.

The spontaneous termination of the tachycardia displayed in Figure 2 showing a typical “cool-down” phenomenon of gradual slowing of the tachycardic rate before cessation. During the rate slowing one can now discern the P waves, which were previously merging with the T wave and could not be clearly distinguished, with progressive lengthening of the PP cycle length. 

Ultimately, observation of the cool down phenomenon elegantly confirms the diagnosis of focal atrial tachycardia, and this is seen in the telemetry strip shown in Figure 2. Cool down and warm up describe a more gradual deceleration/acceleration with changes in heart rate, typically lasting a few seconds. Cool down and warm up are only seen in cases where enhanced automaticity is the underlying cause of the arrhythmia [1,6]. Observing this essentially ruled out arrythmias caused by reentry circuits, including atrial flutter, AVNRT and AVRT, and narrowed the differential to focal atrial tachycardia. 

## 4. Conclusions

Differentiating narrow complex tachycardia diagnoses can often be challenging. Evaluation usually involves two primary components: assessment of the patient for symptoms and signs of hemodynamic stability (or instability), and assessment of the patient’s ECG for clues as to the type of tachycardia present, which shows a heart rate greater than 100 beats per minute along with narrow QRS complexes that are less than 120 milliseconds in duration. Rarely, warm up or cool down phenomenon is seen on ECG or telemetry which gives us a clue as seen in this case. 

## Data Availability

Data available upon request.
